# Risk of antibiotics associated with *Clostridioides difficile* infection: Antibiotic stewardship in action

**DOI:** 10.1017/ash.2022.286

**Published:** 2022-08-26

**Authors:** Andrew T. Dysangco, Tamra M. Pierce, Dawn M. Bravata

**Affiliations:** 1 Department of Veterans’ Affairs (VA) Health Services Research and Development (HSR&D) Precision Monitoring to Transform Care (PRISM) Quality Enhancement Research Initiative (QUERI), Indianapolis, Indiana; 2 VA HSR&D Center for Health Information and Communication (CHIC), Richard L. Roudebush VA Medical Center, Indianapolis, Indiana; 3 Medicine Service, Richard L. Roudebush VA Medical Center, Indianapolis, Indiana; 4 Department of Medicine, Indiana University School of Medicine, Indianapolis, Indiana; 5 Pharmacy Service, Richard L. Roudebush VA Medical Center, Indianapolis, Indiana; 6 Department of Neurology, Indiana University School of Medicine, Indianapolis, Indiana; 7 Regenstrief Institute, Indianapolis, Indiana

## Abstract

We examined risk associated with antibiotics used for *Clostridioides difficile* infection (CDI) at a single site from 2018 through 2020. Overall, 78 patients had nonrecurrent infections. Among inpatient antibiotics, intravenous meropenem had the highest CDI rate (3.56 per 1,000 days of therapy; n = 2 cases). Among outpatient antibiotics, metronidazole had the highest rate (0.071 per 1,000 pills dispensed; n = 3 cases).

*Clostridioides difficile* infection (CDI) is a diarrheal disease commonly associated with antibiotics. CDI caseloads have decreased through antibiotic stewardship, infection prevention,^1^ and more specific testing recommendations.^2^ However, CDI continues to be an avoidable source of morbidity and mortality.^1,3^

As antibiotic stewardship programs (ASPs) seek to avoid high-risk antibiotics (eg, clindamycin, cephalosporins, and fluoroquinolones),^4^ CDI risk shifts to antibiotics that are used. The objectives of this pilot project were to identify the antibiotics associated with CDI in our institution and to estimate the risk of these antibiotics by the amount of antibiotic consumed or prescribed.

## Methods

This retrospective cohort included patients diagnosed with CDI at the Richard L. Roudebush Veterans’ Administration Medical Center from July 2018 to December 2020. CDI cases were identified based on a positive *C. diff* toxin polymerase chain reaction assay, followed by positive glutamate dehydrogenase enzyme-linked immunosorbent assay (EIA) and toxin EIAs based on the recommended multistep testing algorithm.^3^

All nonrecurrent CDIs were included.^3^ Chart review confirmed CDI diagnosis, excluded recurrent infections, and collected demographics, antibiotic exposure, CDI disease severity (nonsevere, severe, and fulminant),^3^ body mass index (BMI), and, over the 30 days prior to diagnosis, proton pump inhibitor (PPI) use, receipt of cancer chemotherapy, and abdominal surgery.

We defined an antibiotic exposure as an antibiotic given or prescribed for ≥3 calendar days within 30 days prior to CDI diagnosis.^5^ We did not combine antibiotics of the same class, nor did we analyze antibiotic combinations or multiple antibiotic exposures. Antibiotics associated with only 1 CDI case were excluded. Inpatient antibiotic days of therapy (DOT) and outpatient number of pills dispensed were used to quantify antibiotic consumption for rate calculations. Oral and liquid preparations rarely used or those with specific niche uses (eg, doxycycline 50 mg, used by the dental service) were excluded.

Descriptive analysis was stratified by CDI severity for antibiotic exposure. Means, frequencies, and rates were calculated using Microsoft 365 Excel software (Microsoft, Redmond, WA). The estimated prevalence of antibiotic use was 76.8% in the 30 days prior to CDI.^5^ On average, 32 CDI diagnoses were made in the institution annually. Given a 2.5-year study period, we anticipated a sample size of 80 CDI patients and 63 antibiotic-associated cases. This study was approved by Indiana University Institutional Review Board.

## Results

In total, 91 CDI cases were diagnosed; 78 were nonrecurrent infections. Of these 78 CDI patients, 75 (96.15%) were male, and the mean patient age was 68.83 years (SD, 11.35) (Table 1). Overall, 51 cases (65.38%) were not severe, 10 cases (12.82%) were severe, and 6 cases (7.69%) were fulminant. Due to lack of laboratory data, the severity of 11 cases (14.10%) could not be assessed; however, all were diagnosed and treated in the outpatient setting.

**Table 1.** Patient Characteristic by *Clostridioides difficile* Disease Severity

Baseline Characteristics	All Cases (N = 78)	Disease Severity			
		Non-Severe (N = 51)	Severe (N = 10)	Fulminant (N = 6)	Unknown (N = 11)
Age, mean y (SD)	68.83 (11.35)	68.23 (12.35)	73.17 (8.54)	70.64 (4.21)	66.68 (10.31)
BMI, mean kg/m^2^ (SD)	28.06 (6.99)	28.25 (6.77)	25.88 (6.71)	31.95 (8.42)	26.85 (6.15)
Chemotherapy, no. (%)	8 (10.26)	7 (13.73)	0	0	1 (9.09)
Abdominal surgery, no. (%)	4 (5.13)	1 (1.96)	3 (30.0)	0	0
PPI use, no. (%)	32 (41.03)	20 (39.22)	7 (70.0)	2 (33.33)	3 (27.27)
Antibiotics, no. (%)^a^	37 (47.44)	25 (49.02)	6 (60.0)	2 (33.33)	4 (36.36)

Note. BMI, body mass index; PPI, proton pump inhibitor.

a Any antibiotic administered or prescribed for at least 3 days during the 30 days preceding *C. difficile* infection diagnosis.

Antibiotics were commonly prescribed in the preinfection period (72 antibiotics exposures): 37 (47.44%) of 78 cases had an antibiotic of ≥3 days duration in the 30 days before CDI (Table 1). The most common antibiotics used prior to CDI were inpatient piperacillin-tazobactam (PTZ) and outpatient amoxicillin-clavulanate (AMC) (Table 2). PTZ was also the most common antibiotic given prior to severe and fulminant CDI cases.

**Table 2.** Antibiotics Given 30 Days Prior to Development of CDI

	Frequency					CDI Rate per 1,000 DOT (CDI/1 KDOT)
Inpatient Antibiotics	Total	Non-severe	Severe	Fulminant	Unknown	
**Intravenous**						
Piperacillin-tazobactam	9	4	3	2	…	1.54
Ceftriaxone	5	4	…	1	…	0.72
Vancomycin	4	3	1	…	…	0.57
Ampicillin-sulbactam	4	3	…	1	…	1.58
Metronidazole	3	3	…	…	…	1.55
Cefazolin	2	2	…	…	…	0.38
Cefepime	2	2	…	…	…	0.52
Meropenem	2	2	…	…	…	3.56
**Oral**						
Trimethroprim-sulfamethoxazole	4	3	1	…	…	2.06
Amoxicillin-clavulanate	3	2	1	…	…	1.16
Azithromycin	2	2	–	…	…	0.50
Cefuroxime	2	2	–	…	…	3.17
Ciprofloxacin	2	1	1	…	…	1.18
Metronidazole	2	1	1	…	…	1.75
**Outpatient oral**						CDI Rate per 1,000 PD (CDI/1 KPD)
Amoxicillin-clavulanate	5	4	…	…	1	0.050
Cephalexin	3	3	…	…	…	0.016
Clindamycin	3	…	…	…	3	0.020
Metronidazole	3	3	…	…	…	0.071
Ciprofloxacin	2	1	1	…	…	0.031

Note. CDI, *Clostridioides difficile* infection; DOT, days of therapy; KDOT 1,000 days of therapy; PD, outpatient number of pills dispensed; KPD, 1,000 pills dispensed.

The highest rate of postantibiotic CDI occurred with inpatient meropenem (MEM; 3.65 CDI per 1 KDOT) and outpatient metronidazole (0.071 CDI per 1 KPD).

## Discussion

In this pilot study, we were able to quantify the CDI risk of antibiotics used in our facility by dividing the number of CDI cases associated with each antibiotic by a measure of antibiotic consumption (DOT for inpatient and number of pills dispensed for outpatient medications). The denominator data were readily available from our pharmacy and through our ASP. These calculations provided an institution-specific risk profile that reflected our antibiotic use pattern and guided our stewardship efforts. Although the risk estimates may not be generalizable to other institutions, the approach provides a simple way to describe institution-specific antibiotic CDI risk. Future studies should validate this approach to measuring CDI risk of antibiotics and should evaluate whether changes in ASP recommendations can shift CDI risk and/or decrease CDI events.

Overall, PTZ was the most common IV antibiotic given prior to CDI (n = 9 cases). We will leverage this finding to encourage more rational PTZ use, especially when anaerobic or antipseudomonal coverage is not indicated. Ceftriaxone (CTX) was associated with 5 CDIs. CTX is one of the most common antibiotics used in the hospital, and the CDI rate with this antibiotic was relatively low (0.72 CDI per 1 KDOT). In diseases for which ampicillin-sulbactam is an alternative to CTX (eg, community-acquired pneumonia), favoring the former may increase CDIs; ampicillin-sulbactam had a higher CDI rate (1.58 CDI per 1 KDOT).

Meropenem was associated with 2 CDI cases but had the highest CDI rate among the IV antibiotics for inpatients (3.56 CDI per 1 KDOT). Although the CDI rate is likely affected by low consumption and use in high-risk (ie, critically ill) patients, our finding was consistent with the prevailing literature.^4,6^ We have limited the use of meropenem to those at risk for extended-spectrum β-lacatmase–producing Enterobacterales, and we are considering opportunities to further reduce inappropriate use. Cefepime had a lower CDI risk than PTZ. Although some data suggest that PTZ is protective of CDI,^6^ our findings suggest that cefepime may be preferentially recommended over PTZ in infections when either is appropriate.

In common diseases like inpatient urinary tract infections, we may recommend de-escalation to oral regimens like AMC or cefuroxime. Cefuroxime had higher CDI risk (3.17 CDI per 1 KDOT) than AMC (1.16 CDI per 1 KDOT). However, it is doubtful that AMC was safer, given that it was associated with 5 outpatient CDI cases. We were unable to combine inpatient and outpatient AMC data because the measures differ. Also, we were unable to determine whether all dispensed antibiotics were consumed. Finally, being an inpatient increases CDI risk.^7^

High-risk antibiotics (cephalosporins, ciprofloxacin, and clindamycin)^4–6^ were also identified as high risk in our study. Some narrow-spectrum and lower-risk antibiotics were also associated with CDI in our study (ie, vancomycin, metronidazole, azithromycin, trimethoprim-sulfamethoxazole), perhaps due to high utilization or their use in combination with higher-risk antibiotics. We did not assess the effects of multiple antibiotic exposures on CDI risk.

Most CDI cases were not severe. Increased age has been associated with increased severity,^8^ consistent with our results. Only 10.26% of cases had chemotherapy prior to CDI, consistent with studies in which no specific chemotherapy has been shown to be an independent risk factor for CDI.^9^ PPIs were commonly used prior to CDI, similar to prior studies.^10^

Our study had several limitations. We considered every antibiotic exposure as an independent exposure, and we did not analyze the effects of combined antibiotics or multiple antibiotics given prior to CDI. We also limited our chart review to our institution’s records; thus, antibiotics prescribed and CDI diagnosed elsewhere were not included. The single-center nature of the study limited generalizability and did not supplant the established antibiotic risk based on larger studies. Although we examined the association between antibiotics and the development of CDI, causality cannot be inferred. The relatively small sample size limited comparisons of CDI risk across antibiotics. We intend to conduct a future study when we have additional years of data and can examine how modified antimicrobial policies may have changed CDI risk for individual antibiotics.

In this single-institution evaluation, simple calculations using readily available data to stratify CDI risk to help guide antibiotic stewardship policies to reduce CDI.

## References

[1] Guh AY , Mu Y , Winston LG , et al. Trends in US burden of *Clostridioides difficile* infection and outcomes. N Engl J Med 2020;382:1320–1330. 32242357 10.1056/NEJMoa1910215 PMC7861882

[2] Lee HS , Plechot K , Gohil S , Le J. *Clostridium difficile*: diagnosis and the consequence of over diagnosis. Infect Dis Ther 2021;10:687–697. 33770398 10.1007/s40121-021-00417-7 PMC8116462

[3] McDonald LC , Gerding DN , Johnson S , et al. Clinical practice guidelines for *Clostridium difficile* infection in adults and children: 2017 update by the Infectious Diseases Society of America (IDSA) and Society for Healthcare Epidemiology of America (SHEA). Clin Infect Dis 2018;66:e1–e48. 29462280 10.1093/cid/cix1085 PMC6018983

[4] Brown KA , Khanafer N , Daneman N , Fisman DN. Meta-analysis of antibiotics and the risk of community-associated *Clostridium difficile* infection. Antimicrob Agents Chemother 2013;57:2326–2332. 23478961 10.1128/AAC.02176-12 PMC3632900

[5] Hensgens MP , Goorhuis A , Dekkers OM and Kuijper EJ. Time interval of increased risk for *Clostridium difficile* infection after exposure to antibiotics. J Antimicrob Chemother 2012;67:742–748. 22146873 10.1093/jac/dkr508

[6] Slimings C , Riley TV. Antibiotics and hospital-acquired *Clostridium difficile* infection: update of systematic review and meta-analysis. J Antimicrob Chemother 2014;69:881–891. 24324224 10.1093/jac/dkt477

[7] Freedberg DE , Salmasian H , Cohen B , Abrams JA , Larson EL. Receipt of antibiotics in hospitalized patients and risk for *Clostridium difficile* infection in subsequent patients who occupy the same bed. JAMA Intern Med 2016;176:1801–1808. 27723860 10.1001/jamainternmed.2016.6193 PMC5138095

[8] Czepiel J , Dróżdż M , Pituch H , et al. *Clostridium difficile* infection: review. Eur J Clin Microbiol Infect Dis 2019;38:1211–1221. 30945014 10.1007/s10096-019-03539-6 PMC6570665

[9] Neemann K , Freifeld A. *Clostridium difficile*–associated diarrhea in the oncology patient. J Oncol Pract 2017;13:25–30. 28084880 10.1200/JOP.2016.018614

[10] Ofori E , Ramai D , Dhawan M , Mustafa F , Gasperino J , Reddy M. Community-acquired *Clostridium difficile*: epidemiology, ribotype, risk factors, hospital and intensive care unit outcomes, and current and emerging therapies. J Hosp Infect 2018;99:436–442. 29410012 10.1016/j.jhin.2018.01.015

